# LURAP1L-AS1 long noncoding RNA promotes breast cancer progression and associates with poor prognosis^⋆^

**DOI:** 10.1016/j.ncrna.2025.01.006

**Published:** 2025-01-19

**Authors:** Radhakrishnan Vishnubalaji, Dania Awata, Nehad M. Alajez

**Affiliations:** a Translational Oncology Research Center (TORC), Qatar Biomedical Research Institute (QBRI), Hamad Bin Khalifa University (HBKU), Qatar Foundation (QF), PO Box 34110, Doha, Qatar; b College of Health & Life Sciences, Hamad Bin Khalifa University (HBKU), Qatar Foundation (QF), PO Box 34110, Doha, Qatar

**Keywords:** LURAP1L-AS1, lncRNA, Breast cancer, TNBC

## Abstract

Long noncoding RNAs (lncRNAs) are emerging as critical regulators of cancer biology, yet their roles in breast cancer, particularly in triple-negative breast cancer (TNBC), remain incompletely understood. Through a custom siRNA library screen targeting TNBC-associated lncRNAs in MDA-MB-231 and BT-549 TNBC cell models, we identified LURAP1L-AS1 as a key modulator of TNBC progression. Survival analysis of TNBC patients demonstrated a significant association between elevated LURAP1L-AS1 expression and poor clinical outcomes.

LURAP1L-AS1 knockdown significantly impaired colony formation and organoid growth of TNBC models, associated with increased apoptosis thus highlighting its role in promoting tumorigenicity. RNA sequencing of LURAP1L-AS1-depleted cells revealed dysregulation of pathways related to cell proliferation, apoptosis, migration, and RNA processing. Bioinformatics analysis predicted LURAP1L-AS1 to function as a competitive endogenous RNA (ceRNA), sponging key microRNAs, such as miR-7a-5p, miR-101-3p, miR-181a-5p, and miR-27a-3p, thereby modulating oncogenes including EZH2, MCL1, and KRAS, which are linked to increased cancer cell survival, proliferation, and metastasis.

In addition to its role in TNBC, correlation analysis using breast cancer patient datasets revealed a significant association between LURAP1L-AS1 and ESR1 expression, suggesting its broader impact across breast cancer subtypes. Concordantly, LURAP1L-AS1 depletion inhibited estrogen receptor-positive (ER+) MCF7 breast cancer cells colony formation and organotypic growth.

Our findings establish LURAP1L-AS1 as a functional lncRNA that promotes breast cancer progression, highlighting its potential for use in RNA-based therapies for breast cancer.

## Graphical abstract

**Figure:**
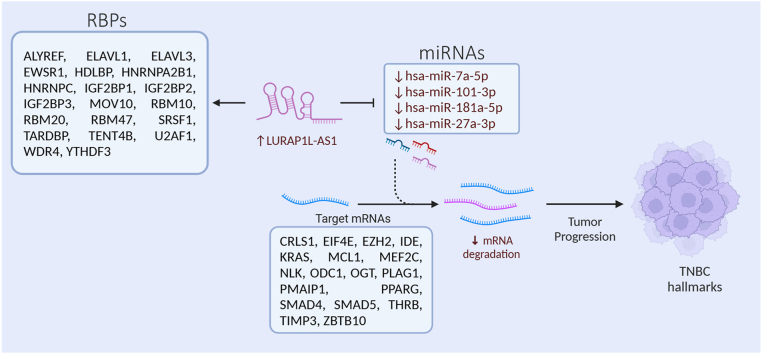

## 1 Introduction

Breast cancer is a heterogeneous disease with diverse molecular profiles and clinical outcomes. Among the various breast cancer subtypes, triple-negative breast cancer (TNBC) is the most aggressive, characterized by limited targeted therapies, resistance to conventional treatments, and high mortality rates [1]. Despite significant progress in breast cancer research, the molecular mechanisms driving TNBC progression remain poorly understood. Recent studies have emphasized the critical role of noncoding RNAs (ncRNAs) in regulating cancer biology, with growing evidence linking them to the regulation of gene expression, tumor growth, metastasis, and therapeutic resistance [2].

Long noncoding RNAs (lncRNAs), a class of ncRNAs longer than 200 nucleotides, have emerged as key regulators in various cellular processes, including transcriptional regulation, RNA processing, and chromatin remodeling [3]. In cancer, lncRNAs have been found to play both tumor-promoting and tumor-suppressing roles, depending on the context. They can act through a variety of mechanisms, including the regulation of gene expression via chromatin remodeling, acting as scaffolds for protein complexes. LncRNAs can also serve as competitive endogenous RNAs (ceRNAs), binding to microRNAs (miRNAs) and preventing them from silencing target genes, including critical oncogenes and tumor suppressors. These complex regulatory networks make lncRNAs potential prognostic biomarkers and therapeutic targets in cancer [4], particularly in aggressive subtypes like TNBC, where targeted treatment options are limited.

Our previous research has demonstrated that certain lncRNAs are associated with poor prognosis in breast cancer, positioning them as potential biomarkers for predicting clinical outcomes [5,6]. Despite growing evidence of their importance in cancer biology, many lncRNAs remain unexplored, particularly in the context of breast cancer. Identifying novel lncRNA targets is crucial for the development of effective, targeted RNA-based therapies, as they offer a new avenue for treating cancers that are resistant to traditional approaches.

Herein, we investigate the role of LURAP1L-AS1 lncRNA in breast cancer, identified through customed siRNA library screen. Our study reveals that LURAP1L-AS1 is overexpressed in both TNBC and estrogen receptor-positive (ER+) breast cancer subtypes, and its elevated expression in TNBC correlates with poor patient survival. Through a combination of siRNA screening, RNA sequencing (RNA-Seq), and functional assays in various cell models, we identified LURAP1L-AS1 as a critical modulator of tumorigenic properties. Our findings demonstrate that LURAP1L-AS1 knockdown leads to a reduction in colony-forming unit (CFU) formation, promotes cell death, alters key tumor-related pathways, and potentially functions as a ceRNA, sponging specific miRNAs to regulate the expression of several oncogenes. This study provides evidence that LURAP1L-AS1 is an important promoter of breast cancer progression, is associated with poor clinical outcomes, and holds potential as a target for novel RNA-based therapies. Given its role in both TNBC and ER+ breast cancer, LURAP1L-AS1 represents a promising candidate for therapeutic intervention, offering new venue for more effective treatments for breast cancer patients.

## 2 Material and methods

### 2.1 Cell culture and transfection

The human TNBC cell lines, MDA-MB-231 and BT-549, were cultured in Dulbecco's Modified Eagle Medium (DMEM) supplemented with 10 % fetal bovine serum (FBS) and 1 % penicillin/streptomycin (Pen-Strep) (Thermo Scientific, Rockford, IL, USA). Cells were maintained in a humidified incubator at 37 °C with 5 % CO₂. To assess the functional role of various lncRNAs in the library, TNBC cells were reverse transfected using Lipofectamine 2000 (Invitrogen), following the method previously described [6]. Briefly, siRNAs (30 nM) and 1.5 μL of Lipofectamine 2000 were separately diluted in 50 μL Opti-MEM (Gibco, Carlsbad, CA, USA), mixed, and incubated for 20 min at room temperature. This siRNA-lipid complex was then added to 300 μL of cell suspension (0.168 × 10⁶ cells/mL) and incubated for 20 h, after which the medium was replaced with complete DMEM.

### 2.2 Colony forming unit (CFU) assay

SiRNA transfection was performed with 200 μL of transfection cocktail and 600 μL of cell suspension (0.1 × 10⁶ cells/600 μL/well) in a 12-well plate. The clonogenic potential of siRNA-transfected TNBC cells was evaluated using a CFU assay on day 7 post transfection. Colonies were stained with 0.5 % crystal violet for 10 min with continuous shaking, followed by washing three times with tap water. After air drying, colonies were imaged and quantified. Crystal violet staining in 10 % SDS was used to measure absorbance at 590 nm. The experiment was performed in biological duplicates with four technical replicates, and results are presented as mean ± SD.

### 2.3 Viability staining

To assess cell viability, acridine orange/ethidium bromide (AO/EtBr) dual staining was performed 5 days post-transfection. Cells were washed with PBS and stained with a solution containing 100 μg/mL AO and 100 μg/mL EtBr (Sigma-Aldrich, St. Louis, MO, USA). Fluorescent images were captured using an Olympus IX73 fluorescence microscope (Olympus, Tokyo, Japan), with AO marking all cells, while EtBr staining dead cells.

### 2.4 Organoid culture

For 3D organoid formation, siRNA-transfected cells were pelleted and mixed (30,000 cells/100 μL) with Matrigel (Corning; Growth Factor Reduced Basement Membrane Matrix) at 4 °C as previously described [6]. The cell-Matrigel mixture was plated as droplets on pre-warmed ultra-low attachment culture dishes (Corning). After solidification at 37 °C for 20 min, an expansion medium was added. Organoids were monitored under a microscope for one week.

### 2.5 Validation using breast cancer datasets

RNA-Seq data were obtained from 360 TNBC (accession no. PRJNA486023) using the SRA toolkit v2.9.2 [6,7] followed by transcriptomic profiling using GENCODE release (v33) and KALLISTO 0.4.2.1 as described before [8,9]. Normalized expression values (TPM, transcript per million) from the 360-patient TNBC cohort were imported into iDEP.96. Differential expression and gene set enrichment analysis were performed using iDEP.96. Significantly differentially expressed genes (DEGs) were identified using a cutoff of 1.5-fold change (FC) and FDR <0.1, and further analyzed using Ingenuity Pathway Analysis (IPA) for pathway enrichment and miRNA-mRNA interaction networks. Survival analyses were conducted using the Kaplan-Meier method in IBM SPSS Statistics version 29.0.1.0. TNBC samples were classified into four transcriptome-based subtypes: luminal androgen receptor (LAR), immunomodulatory (IM), basal-like immune-suppressed (BLIS), and mesenchymal (MES) as reported by Jiang et al. [8]. LURAP1L-AS1 expression in TNBC (n = 42), ER+ (n = 42), and normal breast tissue (n = 56) from the PRJNA251383 were assessed using KALLISTO 0.4.2.1 and GENCODE release (v33) annotations. Correlation between LURAP1L-AS1 and ESR1 in breast cancer was assessed using StarBase database [10].

### 2.6 Total RNA sequencing (RNA-seq) from LURAP1L-AS1-depleted TNBC cells

MDA-MB-231 cells were transfected with siRNAs targeting LURAP1L-AS1 or a scrambled control siRNA (Dharmacon ON-TARGETplus, Thermo Scientific). After 72 h, total RNA was extracted and subjected to library preparation employing the TruSeq Stranded Total RNA Library Kit (Illumina). Sequencing libraries were prepared, and paired-end reads were aligned to the GRCh38 reference genome using the CLC Genomics Workbench v21.0.5. DEGs were analyzed with iDEP.96 as we described before [11].

### 2.7 Bioinformatics analysis of LURAP1L-AS1-associated miRNA and RBP networks

To identify potential miRNA and gene targets for the LURAP1L-AS1 circuit, we utilized the lncBase v3.0 database (https://diana.e-ce.uth.gr/lncbasev3) to compile a list of miRNAs interacting with LURAP1L-AS1 based on HITS-CLIP data [12]. This list, in conjunction with downregulated genes identified in LURAP1L-AS1-depleted TNBC cells, was imported into IPA. The microRNA Target Filter in IPA was then used to identify miRNA-mRNA networks, where only experimentally validated and predicted interactions were included in the final analysis. To construct the LURAP1L-AS1-RBP network, we retrieved the list of LURAP1L-AS1-interacting RBPs from the ENCORI database (https://rnasysu.com/encori/) [10]. The network was visualized and constructed using Cytoscape v3.10 as described before [13].

### 2.8 Real-Time Quantitative PCR (RT-qPCR)

Gene expression was validated by RT-qPCR using the QuantStudio 7/6 Flex System (Applied Biosystems). Total RNA (500 ng) was reverse-transcribed using the High-Capacity cDNA Reverse Transcription Kit (Applied Biosystems). Reactions were performed with PowerUp™ SYBR™ Green Master Mix and specific primers for LURAP1L-AS1, SEMA3, THRB, EPS8, INPP4B, GPD1L, and ENPP1, while GAPDH served as the internal control. Table 1 lists the sequences of all primers used in current study. Relative gene expression was calculated using the ΔCT method normalized to GAPDH.

**Table 1:** SYBR green primer sequences used in current study.

S.No	Names	Forward Sequences	Reverse Sequences
1	LURAP1L-AS1	ACTCCCCATATTCACAGCAGA	GTCTTCCAGGGCTGCAATTT
2	SEMA3	ACTCAGAGGACCGGGAAGAA	TCCAAGGGAAAATACCGAGGTT
3	THRB	TTTCATCTGCCCCCAGTGAC	GGCTGTAAGGCCATTTTCTGTC
4	EPS8	CGACGCCCTGAGGATGATTT	GGCTGCCACTCGACTTCTAA
5	INPP4B	ACGCAGGAAAGTCAGGCTAA	GCTGGTTTCAAGGCTCTTTGTC
6	GPD1L	ATGCAGACCTGCTGGTGTTT	GCCCTCGTCTATGCCCTTG
7	ENPP1	GGCATGGAACAAGGCAGTTG	TTGGTTCCCGGCAAGAAAGA
8	GAPDH	5′GGAGCGAGATCCCTCCAAAAT3′	5′GGCTGTTGTCATACTTCTCATGG3′

### 2.9 Statistical analysis

DEG analysis was performed using iDEP.96, applying a threshold of ≥2.0-fold change and FDR <0.05, unless stated otherwise. Kaplan-Meier survival curves and log-rank tests were conducted in IBM SPSS v29.0.1.0, with p-values <0.05 considered significant. Survival curve plots were generated using GraphPad Prism v10. All other statistical analyses, including *t*-tests, ANOVA, and data visualization, were conducted using GraphPad Prism v10. Results are presented as mean ± S.E. from at least two independent experiments, with statistical significance set at p < 0.05.

## 3 Results

### 3.1 siRNA library screening identifies LURAP1L-AS1 as a key lncRNA promoting TNBC viability

**Fig. 1:**
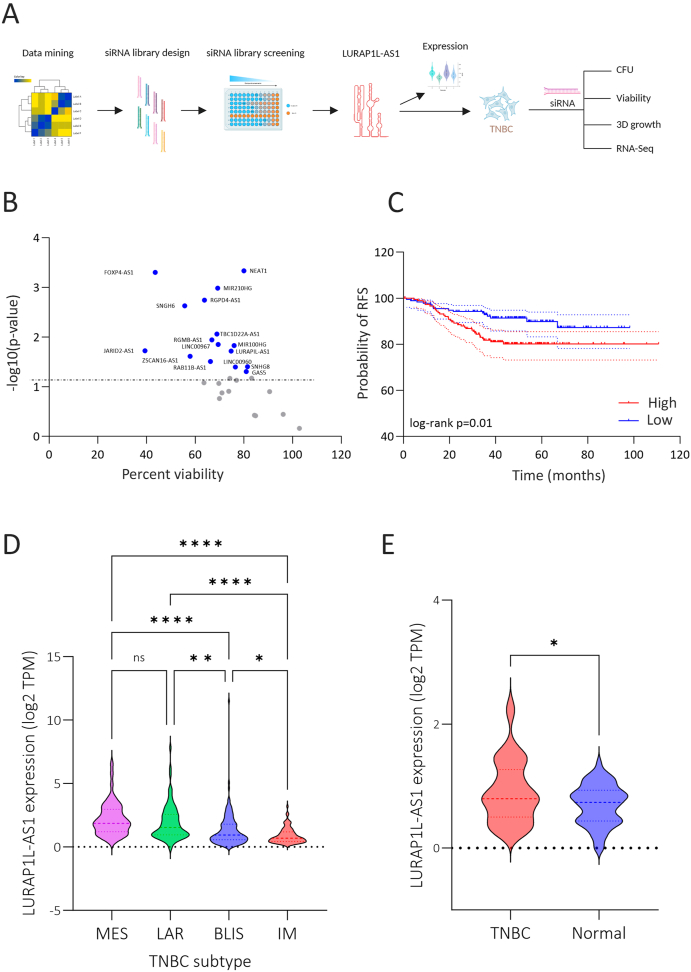
**SiRNA library screen identifies LURAP1L-AS1 as a TNBC-associated lncRNA that promotes TNBC survival and correlates with poor prognosis. (A)** Overview of the experimental workflow using siRNA targeting TNBC associated lncRNAs. **(B)** Volcano plot displaying cell viability (%) on the x-axis and -log10(p-value) on the y-axis from the screen of a custom siRNA library against various lncRNAs in MDA-MB-231 and BT-549 cell lines. Blue circles denote candidates with significantly reduced cell viability. **(C)** Kaplan-Meier survival analysis of 360 TNBC patients stratified by median LURAP1L-AS1 expression (high vs. low). Statistical significance was assessed using the log-rank test. **(D)** Violin plot depicting LURAP1L-AS1 expression across TNBC molecular subtypes (MES, LAR, BLIS, IM) from the PRJNA486023 dataset. ∗p < 0.05, ∗∗p < 0.005, ∗∗∗∗p < 0.00005. **(E)** LURAP1L-AS1 expression levels in TNBC (n = 42) versus normal breast tissue (n = 56) from the PRJNA251383 dataset. ∗p < 0.05.

In this study, a targeted lncRNA screen using a customized siRNA library (Fig. 1A) was performed to assess the impact of a set of TNBC-associated lncRNAs, including JARID2-AS1, FOXP4-AS1, SNGH6, ZSCAN16-AS1, RGPD4-AS1, RAB11B-AS1, RGMB-AS1, TBC1D22A-AS1, LINC00967, MIR210HG, LURAP1L-AS1, MIR100HG, LINC00960, NEAT1, GAS5, and SNHG8, on cell viability in the MDA-MB-231 and BT-549 TNBC models. The library was designed based on our previous investigations on lncRNAs expression and prognosis in TNBC as well as based on literature search. Depletion of several lncRNAs led to reduced cell proliferation, as shown in the volcano plot (Fig. 1B). Notably, we recently reported that LINC00960 promotes TNBC progression [6], while other lncRNAs in the library have been implicated in tumor-promoting roles across various cancer types [[14], [15], [16], [17]]. Among the screened lncRNA candidates, LURAP1L-AS1 emerged as a prominent candidate, demonstrating a significant reduction in cell viability across both TNBC cell lines. Importantly, there are no prior functional studies on the role of LURAP1L-AS1 in TNBC, highlighting its novel relevance in this context.

Further analysis of a TNBC patient cohort (n = 360) demonstrated that high LURAP1L-AS1 (ENSG00000235448) expression correlates with poor relapse free survival (RFS), (Fig. 1C). Interestingly, the highest LURAP1L-AS1 expressions were observed in the MES, LAR, BLIS subtypes relative to the IM subtype (Fig. 1D). Examination of LURAP1L-AS1 in a second breast cancer dataset confirmed elevated LURAP1L-AS1 expression in TNBC (n = 42) compared to normal breast tissue (n = 56), thus suggesting therapeutic window (Fig. 1E).

### 3.2 LURAP1L-AS1 knockdown impairs tumorigenic potential of TNBC cells

**Fig. 2:**
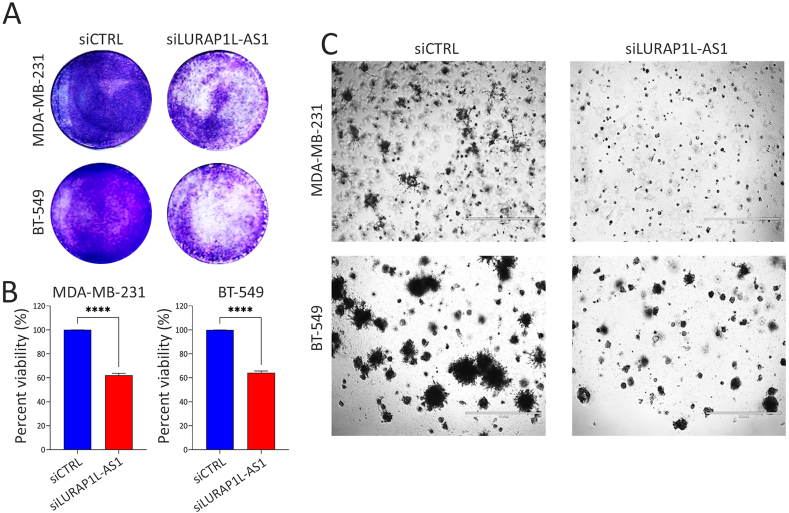
**LURAP1L-AS1 knockdown inhibits colony and organoid formation. (A)** Representative colony formation (CFU) assay showing the impact of LURAP1L-AS1 knockdown on CFU potential in MDA-MB-231 (top) and BT-549 (bottom) cells compared to siControl (siCTRL). (**B)** Qualifications of CFU formation in siCTRL and siLURAP1L-AS1 MDA-MB-231 and BT-549 cells. Data are presented as mean ± S.E.M., relative to siCTRL cells; n = 10. ∗∗∗∗p < 0.00005. **(C)** Reduced organoid formation in MDA-MB-231 (top) and BT-549 (bottom) cells transfected with siLURAP1L-AS1 compared to siCTRL cells.

**Fig. 3:**
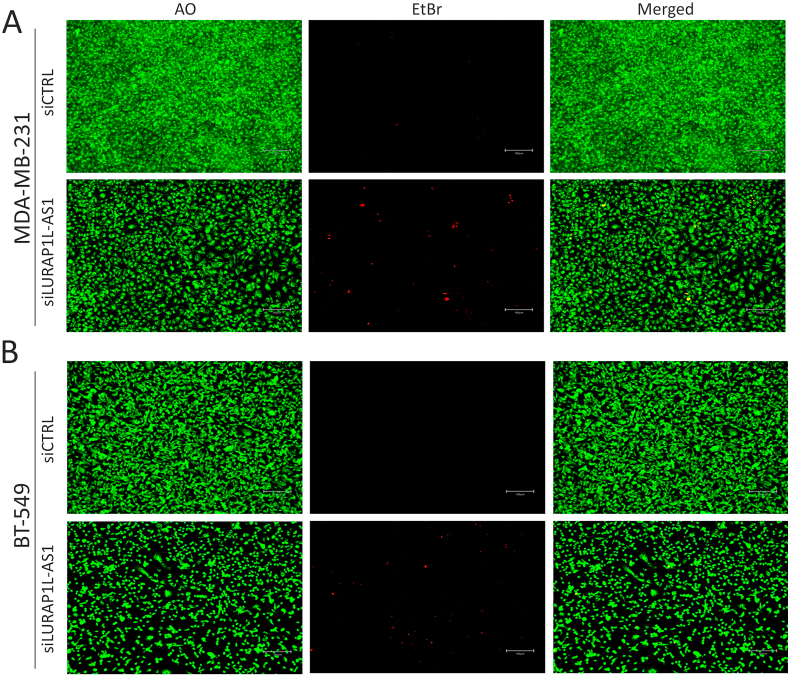
**Live and dead staining in TNBC models following LURAP1L-AS1 suppression.** Representative images showing nuclear staining (green, acridine orange) and dead cell staining (red, ethidium bromide) in MDA-MB-231 **(A)** and BT-549 **(B)** cells after LURAP1L-AS1 knockdown. Scale bar = 100 μm.

To investigate the functional relevance of LURAP1L-AS1 in tumorigenicity, we performed several functional impairment studies in TNBC models using siRNA targeting the LURAP1L-AS1. Both cell models showed significant knockdown of LURAP1L-AS1 expression following siRNA-mediated gene silencing (Fig. S1). CFU assay in MDA-MB-231 and BT-549 cells showed significant reductions in colony formation following LURAP1L-AS1 knockdown (Fig. 2A and B). Concordantly, substantial inhibition of organoid formation and stellate-like cell morphology formation was observed in LURAP1L-AS1-depleted compared to siCTRL cells (Fig. 2C). Viability staining revealed reduced cell growth and increased cell death in MDA-MB-231 (Fig. 3A) and BT-549 (Fig. 3B) cells upon LURAP1L-AS1 suppression, highlighting its critical role in TNBC cell growth and survival.

### 3.3 LURAP1L-AS1 expression and function in ER+ breast cancer

**Fig. 4:**
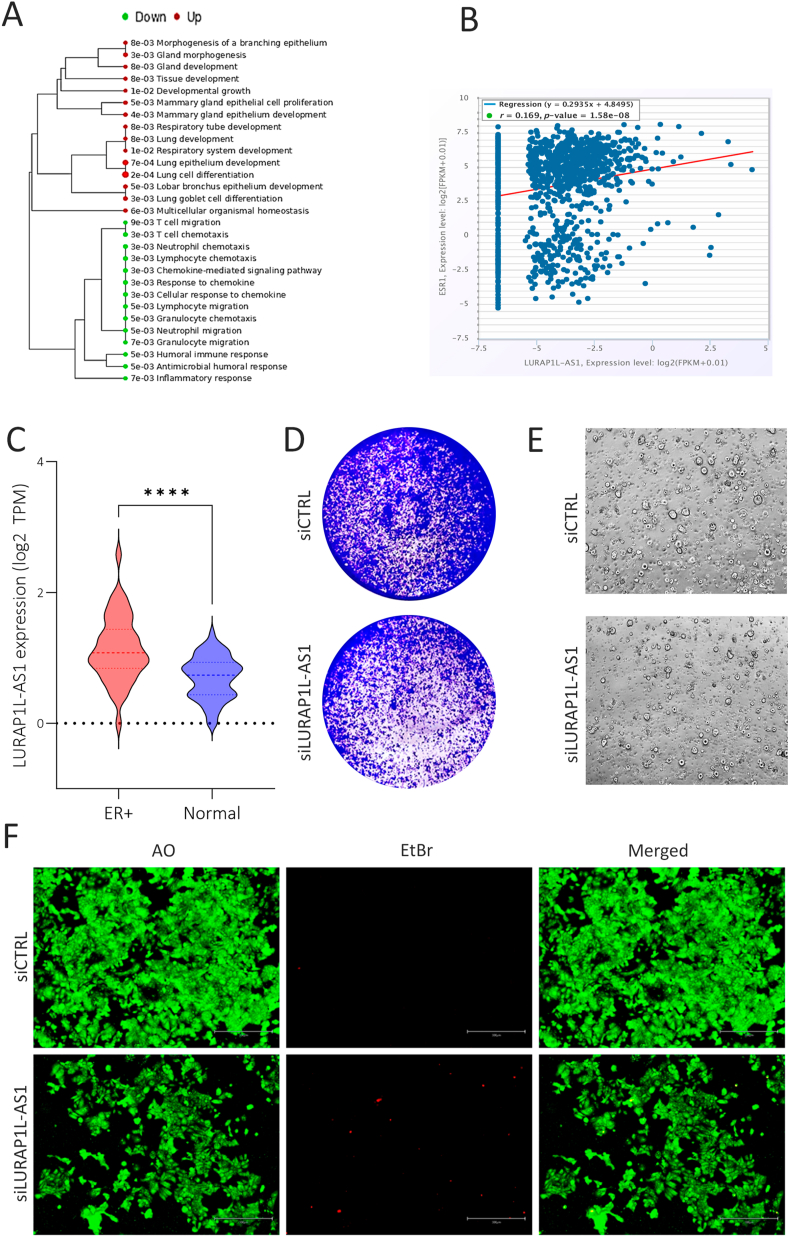
**LURAP1L-AS1 expression and functional impact in ER+ breast cancer. (A)** Gene Ontology (GO) enrichment tree based on differentially expressed genes in LURAP1L-AS1^high^ (n = 180) vs. LURAP1L-AS1^low^ (n = 180) TNBC from the PRJNA486023 dataset. **(B)** Correlation analysis between LURAP1L-AS1 and ESR1 expression in 1104 breast cancer tissues from StarBase database. **(C)** Violin plot depicting LURAP1L-AS1 expression in ER+ breast cancer tissue (n = 42) compared to normal breast tissue (n = 56) from the PRJNA251383 dataset. ∗∗∗∗p < 0.00005. **(D)** CFU assay demonstrating the effect of LURAP1L-AS1 knockdown on colony formation in MCF7 cells. **(E)** Reduced organotypic formation in MCF7 cells transfected with siLURAP1L-AS1 compared to siCTRL. **(F)** Live and dead cell staining of MCF7 cells following LURAP1L-AS1 knockdown (Scale bar = 100 μm).

To gain deeper insight into the functional consequences of LURAP1L-AS1 expression, we performed pathway enrichment analysis using differentially expressed protein-coding genes between LURAP1L-AS1^high^ and LURAP1L-AS1^low^ TNBC subgroups. Gene Ontology (GO) enrichment analysis highlighted pathways associated with mammary gland development and epithelial cell proliferation in the LURAP1L-AS1^high^ subgroup, suggesting a potential role in organ development. Conversely, pathways related to granulocyte and agranulocyte migration in response to chemical stimuli and chemokine-mediated signaling were notably suppressed in this subgroup (Fig. 4A). To confirm the clinical relevance of our findings, we analyzed LURAP1L-AS1 and ESR1, key regulator of mammary development, co-expression levels in a large breast cancer cohort (n = 1104) using the StarBase database. The data in (Fig. 4B) showed a significant positive correlation between LURAP1L-AS1 and ESR1 expression in breast cancer patients (r = 0.169, p = 1.58 × 10⁻⁸), underscoring LURAP1L-AS1 as a clinically relevant marker in ER+ breast cancer. Concordantly, significant upregulation in LURAP1L-AS1 expression was observed in ER+ breast cancers compared to normal breast tissue, as shown in Fig. 4C.

The functional impact of LURAP1L-AS1 was further validated in both 2D and 3D cultures of MCF7 (ER+) cells. In agreement with our findings in TNBC models, LURAP1L-AS1 knockdown in MCF7 cells resulted in a significant reduction in colony formation and organotypic growth (Fig. 4D and E). Additionally, viability staining of siLURAP1L-AS1-treated MCF7 cells demonstrated decreased cell proliferation and increased cell death compared to siCTRL cells (Fig. 4F).

### 3.4 LURAP1L-AS1 functions as a competing endogenous RNA (ceRNA)

**Fig. 5:**
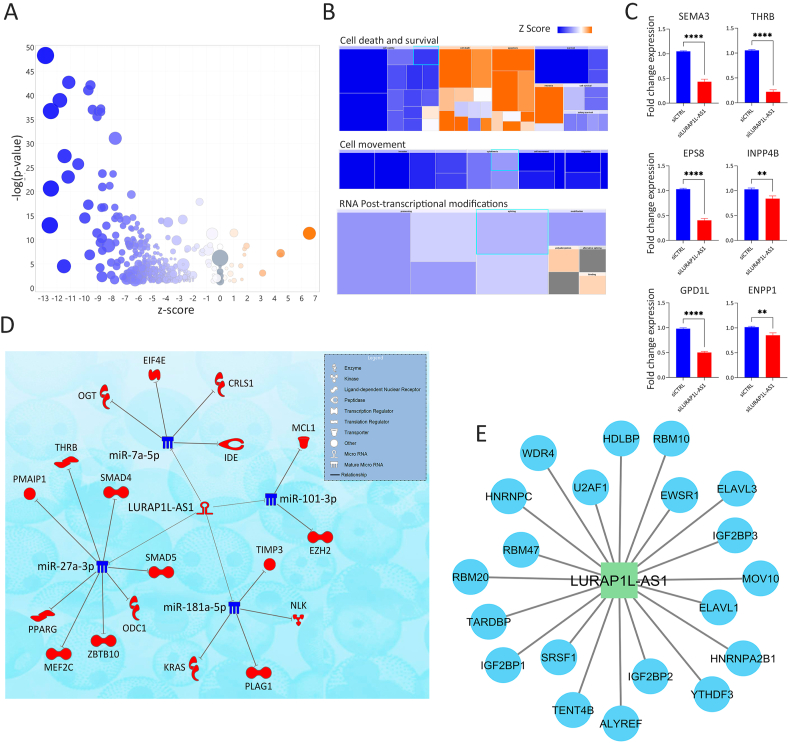
**Functional impact of LURAP1L-AS1 depletion in TNBC. (A)** Volcano bubble plot illustrating activated (orange) and suppressed (blue) canonical pathways in LURAP1L-AS1-depleted TNBC cells, based on RNA-Seq analysis. The x-axis represents the z-score, while the y-axis represents -log(p-value). **(B)** Disease and function pathway map showing activated (orange) and suppressed (blue) categories in LURAP1L-AS1-knockdown TNBC cells based on IPA analysis. **(C)** Validation of selected target genes identified from RNA-Seq. Data are presented as mean ± S.E.M., n = 9. ∗∗p < 0.005, ∗∗∗∗p < 0.00005. **(D)** Schematic representation of the ceRNA mechanism involving LURAP1L-AS1. Upregulated LURAP1L-AS1 (shown in red) in breast cancer cells leads to sponging of miR-7a-5p, miR-101-3p, miR-181a-5p, and miR-27a-3p (shown in blue) leading to upregulation of oncogenic gene targets(shown in red). **(E)** Analysis of the ENCORI database reveals that LURAP1L-AS1 potentially exerts its function through interactions with a number of RNA-binding proteins (RBPs), including ALYREF, ELAVL1, ELAVL3, EWSR1, HDLBP, HNRNPA2B1, HNRNPC, IGF2BP1, IGF2BP2, IGF2BP3, MOV10, RBM10, RBM20, RBM47, SRSF1, TARDBP, TENT4B, U2AF1, WDR4, and YTHDF3. These interactions are supported by CLIP-seq data**.**

To investigate the role of LURAP1L-AS1 in TNBC, we performed RNA-Seq analysis to assess the functional impact of its knockdown in MDA-MB-231 cells. RNA-Seq results revealed the downregulation of genes associated with cell cycle checkpoints, mitosis, and oxidative phosphorylation, as shown in the canonical pathway analysis using (IPA, Fig. 5A and Table S1). Further, disease and function analyses indicated reduced cell viability, survival, movement, and RNA post-transcriptional modifications, while pathways related to cell death, apoptosis, and necrosis were upregulated (Fig. 5B). Validation employing RT-qPCR confirmed the differential expression of selected genes, including SEMA3, THRB, EPS8, INPP4B, GPD1L, and ENPP1, which are linked to oxidative phosphorylation in siLURAP1L-AS1 MDA-MB-231 cells (Fig. 5C).

**Fig. 6:**
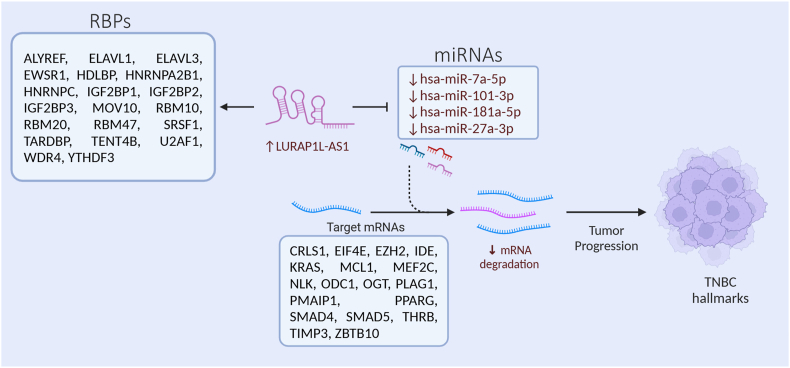
**Multifaceted role of LURAP1L-AS1 in tumorigenesis of breast cancer.** LURAP1L-AS1 potentially contributes to cancer progression by functioning as ceRNA for key tumor suppressor miRNAs, resulting in the upregulation of their target tumor-promoting mRNAs. Furthermore, it interacts with critical RNA-binding proteins (RBPs), thereby influencing various cancer hallmarks.

We subsequently sought to reveal the mechanism by which LURAP1L-AS1 promotes breast cancer tumorigenesis. Interrogation of the lncBase v3.0 database identified the list of miRNAs interacting with LURAP1L-AS1 based on HITS-CLIP data. This list, in conjunction with downregulated genes identified in LURAP1L-AS1-depleted TNBC cells, was imported into IPA. The microRNA Target Filter in IPA was then used to identify miRNA-mRNA networks, where only experimentally validated and predicted interactions were included in the final analysis. Our analysis suggested LURAP1L-AS1 to act as a molecular sponge for miR-7a-5p, miR-101-3p, miR-181a-5p, and miR-27a-3p, resulting in the upregulation of EZH2, MCL1, and KRAS, among other tumor promoting (Fig. 5D). Interrogation of the ENCORI database, LURAP1L-AS1 was also predicted to function through interaction with ALYREF, ELAVL1, ELAVL3, EWSR1, HDLBP, HNRNPA2B1, HNRNPC, IGF2BP1, IGF2BP2, IGF2BP3, MOV10, RBM10, RBM20, RBM47, SRSF1, TARDBP, TENT4B, U2AF1, WDR4, YTHDF3 RNA-binding proteins (RBPs), supported by CLIP-seq data (Fig. 5E). These findings underscore the multifaceted role of LURAP1L-AS1 in TNBC, where it likely contributes to tumorigenesis by acting as a ceRNA for key oncogenic miRNAs and interacting with critical RBPs, thereby influencing gene expressions associated with cell cycle regulation, survival, and metabolism (Fig. 6).

## 4 Discussion

A growing body of evidence has established the crucial role of dysregulated lncRNAs in cancer onset and progression [18]. Our study identifies LURAP1L-AS1 as a pivotal regulator in breast cancer, particularly in TNBC and ER+ subtypes.

Through a targeted siRNA panel screening, we found LURAP1L-AS1 to be essential for TNBC survival. LURAP1L-AS1 exhibited significant upregulated expression in TNBC, with elevated expression correlating with poor prognosis. Functional investigations confirmed that LURAP1L-AS1 is critical for TNBC cell proliferation and survival, as evidenced by substantial reductions in colony formation, organoid formation, and increased cell death upon its knockdown. These findings position LURAP1L-AS1 as a key player in TNBC tumorigenesis.

While our focus was on breast cancer, LURAP1L-AS1 has previously been implicated in the LURAP1L/IKK/IκB/NF-κB signaling pathway in other cancers, such as oral squamous cell carcinoma (OSCC) [19]. In OSCC, LURAP1L-AS1 was shown to facilitate the transformation of fibroblasts into cancer-associated fibroblasts (CAFs) via the PDGF-BB signaling axis, contributing to tumor microenvironment modulation. Although this specific pathway was not the focus of our study, it highlights the potential broader impact of LURAP1L-AS1 in cancer biology and warrants further investigation in breast cancer.

Our RNA-Seq analysis following LURAP1L-AS1 knockdown in TNBC models revealed significant dysregulation in pathways related to cell cycle progression, oxidative phosphorylation, and apoptosis. Pathway enrichment analysis suggests that LURAP1L-AS1 may function as a competing endogenous RNA (ceRNA), sequestering tumor-suppressive miRNAs such as miR-7a-5p, miR-101-3p, miR-181a-5p, and miR-27a-3p. This sponging effect likely upregulates oncogenes, including EZH2, MCL1, and KRAS, thereby promoting tumorigenic signaling and aggressive phenotypes in TNBC.

This ceRNA mechanism is consistent with the role of other lncRNAs in cancer. For instance, our previous investigation suggested LINC00960 to promote breast cancer via the hsa-miR-34a-5p, hsa-miR-16-5p, BMI1, KRAS, and AKT3 network [6]. These findings underscore the critical function of lncRNAs in post-transcriptional regulation, highlighting their potential as therapeutic targets.

Interestingly, our data also point to a broader role for LURAP1L-AS1 in ER+ breast cancer. Knockdown experiments in MCF7 (ER+) cells resulted in reduced colony formation and organotypic growth and increased cell death, indicating its involvement in ER+ breast cancer cell viability. Notably, correlation analyses demonstrated a significant positive association between LURAP1L-AS1 and ESR1 expression, suggesting a possible role for LURAP1L-AS1 in ER+ breast cancer.

While endocrine therapy (ET) remains the cornerstone of treatment for ER+ breast cancer, resistance to ET is a persistent clinical challenge. Mechanisms contributing to ET resistance include ESR1 mutations, fusions, and lncRNA-mediated signaling alterations. Notably, lncRNAs like HOTAIR and TMPO-AS1 have been shown to interact with the ERα protein and modulate estrogen signaling, driving resistance to ET [20,21]. Given the positive correlation between LURAP1L-AS1 and ESR1 expression, our findings raise the possibility that LURAP1L-AS1 could play a role in ET resistance, although the exact mechanisms remain to be elucidated.

Our findings position LURAP1L-AS1 as a potential prognostic marker, particularly in aggressive TNBC subtypes. Its significant association with poor clinical outcomes in TNBC patients suggests that targeting LURAP1L-AS1 could be an alternative strategy for reducing tumor growth and improving patient survival. Additionally, the ceRNA network involving LURAP1L-AS1 and miRNAs presents a novel axis for therapeutic intervention.

Future studies should explore the therapeutic potential of LURAP1L-AS1 inhibitors and their efficacy in preclinical breast cancer models. Furthermore, dissecting the interaction between LURAP1L-AS1 and ER signaling pathways could offer insights into overcoming ET resistance in ER+ breast cancer.

## 5 Conclusions

Our study highlights LURAP1L-AS1 as a key lncRNA promoting tumorigenicity in both TNBC and ER+ breast cancer through multiple regulatory mechanisms. The ceRNA-mediated modulation of oncogenic pathways by LURAP1L-AS1 underscores its potential as a therapeutic target. Further investigations are warranted to fully understand the clinical relevance of LURAP1L-AS1 and to develop RNA-based therapies for breast cancer treatment.

## CRediT authorship contribution statement

**Radhakrishnan Vishnubalaji:** Writing – original draft, Formal analysis, Data curation. **Dania Awata:** Data curation. **Nehad M. Alajez:** Writing – review & editing, Supervision, Funding acquisition, Formal analysis, Conceptualization.

## Ethical approval

Not applicable.

## Declaration of competing interest

The authors declare that they have no known competing financial interests or personal relationships that could have appeared to influence the work reported in this paper.
