# Supplementary material for: LURAP1L-AS1 long noncoding RNA promotes breast cancer progression and associates with poor prognosis
Source: Noncoding RNA Res. 2025 Jan 19;12:1–9. doi: 10.1016/j.ncrna.2025.01.006 (PMC11847224; doi:10.1016/j.ncrna.2025.01.006)
Supplement: Multimedia component 1 [file mmc1.pdf]

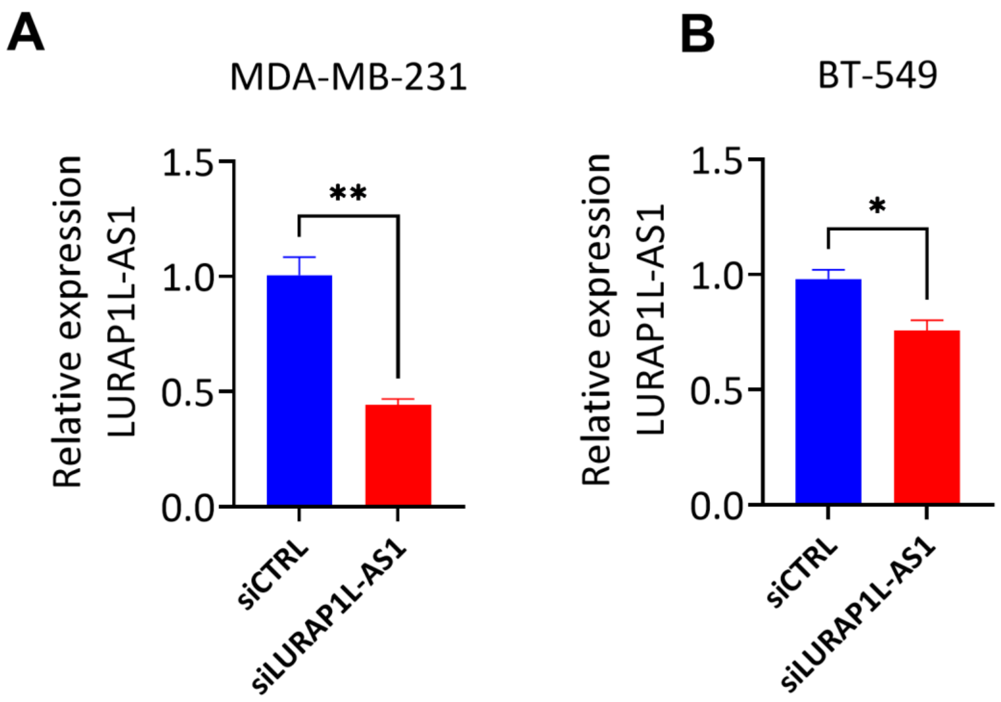

Figure S1. Significant suppression of LURAP1L-AS1 expression in TNBC models. Representative RT-qPCR demonstrating significant suppression of LURAP1L-AS1 expression in MDA-MB-1231 **(A)** or BT-549 **(B).** Data are presented as mean ± S.E.M., n=3. * $p < 0.05$; ** $p < 0.005$.
